# Expression of inhibitory regulators of innate immunity in patients with active tuberculosis

**DOI:** 10.1186/s12879-015-0833-z

**Published:** 2015-02-26

**Authors:** Dana C Blok, Liesbeth M Kager, Arie J Hoogendijk, Ivar O Lede, Wahid Rahman, Rumana Afroz, Paul Bresser, Jaring S van der Zee, Aniruddha Ghose, Caroline E Visser, Menno D de Jong, Abu Shahed Md Zahed, Md Anwar Husain, Khan Mashrequl Alam, Pravat Chandra Barua, Mahtabuddin Hassan, Ahmed Hossain, Md Abu Tayab, Rene Lutter, Nick Day, Arjen M Dondorp, Alex F de Vos, Cornelis van ’t Veer, Tom van der Poll

**Affiliations:** Center for Infection and Immunity Amsterdam (CINIMA), Academic Medical Center/ University of Amsterdam, Amsterdam, The Netherlands; Center for Experimental and Molecular Medicine (CEMM), Academic Medical Center/ University of Amsterdam, Amsterdam, The Netherlands; Division of Infectious Diseases, Academic Medical Center/ University of Amsterdam, Amsterdam, The Netherlands; Department of Microbiology, Academic Medical Center/ University of Amsterdam, Amsterdam, The Netherlands; Department of Pulmonology, Academic Medical Center/ University of Amsterdam, Amsterdam, The Netherlands; Department of Experimental Immunology, Onze Lieve Vrouwe Gasthuis, Amsterdam, The Netherlands; Department of Pulmonology, Chittagong Medical College and Hospital (CMCH), Chittagong, Bangladesh; Department of Internal Medicine, Chittagong Medical College and Hospital (CMCH), Chittagong, Bangladesh; Department of Microbiology, Chittagong Medical College and Hospital (CMCH), Chittagong, Bangladesh; National TB Control and Leprosy Elimination Program, Dhaka, Bangladesh; Chest Disease Clinic Chittagong (CDCC), Chittagong, Bangladesh; Chittagong General Hospital, Chittagong, Bangladesh; Mahidol Oxford Tropical Research Unit, Mahidol University, Bangkok, Thailand

**Keywords:** Innate immunity, Toll-like receptors, Negative regulators of TLR signaling, *mycobacterium tuberculosis*, Lung inflammation, Bronchoscopy

## Abstract

**Background:**

Tuberculosis (TB) is an important cause of morbidity and mortality worldwide. Toll-like-receptors (TLRs) are important for the recognition of the causative agent *Mycobacterium tuberculosis*. Negative regulation of TLRs is necessary to control deleterious inflammatory damage, but could provide a means of immune evasion by *M. tuberculosis* as well.

**Methods:**

To obtain insight in the extent of expression of inhibitory regulators of immunity in patients with active TB, peripheral-blood-mononuclear-cells (PBMCs) and plasma were obtained from 54 TB patients and 29 healthy blood donors from Chittagong, Bangladesh. Bilateral alveolar macrophages were obtained from an infected versus a contralateral normal lung segment of 9 patients. Statistical analyses were performed using Mann–Whitney *U* and Wilcoxon matched pairs testing. Correlations were calculated using the Spearman rho test.

**Results:**

PBMCs harvested from TB patients demonstrated increased mRNA expression of IL-1-receptor-associated-kinase-M, suppressor-of-cytokine-signalling-3 and Toll-interacting-protein. Flow cytometry revealed enhanced expression of IL-1-receptor-like-1 (ST2) on lymphocytes. Plasma soluble ST2 was elevated in patients with TB and correlated with established TB biomarkers, most strongly with soluble interleukin-2 receptor subunit α and interleukin-8. Alveolar macrophage mRNA expression of negative TLR regulators did not differ between the infected and contralateral lung side.

**Conclusion:**

These results show enhanced expression of distinct negative regulators of innate immunity in PBMCs of patients with TB and identify plasma soluble ST2 as a potential novel biomarker for TB disease activity.

## Background

*Mycobacterium tuberculosis* (*Mtb*) remains the leading bacterial agent causing death in humans in the world today [1,2]. Estimations suggest that this acid-fast rod shaped bacillus has infected approximately one third of the world’s population. In 2012 infection developed into active tuberculosis (TB) in 8.6 million cases, resulting in 1.3 million deaths worldwide [1,2]. Bangladesh is one of the most affected countries with 225 new cases per 100,000 inhabitants in 2012 and an overall mortality rate of 45 per 100,000 [1].

Toll-like receptors (TLRs) comprise a family of pattern recognition receptors specifically designed to recognize broadly conserved pathogen associated molecular patterns expressed by microorganisms [3]. While the especially thick cell wall of *Mtb* protects to a certain degree against host immune detection, it also harbors specific antigens able to induce a TLR response [4,5]. Mycobacterial cell wall glycolipids like 19kD lipoprotein, phosphatidylinositol mannoside and lipoarabinomannan have been shown to activate TLR2 signaling (when heterodimerized with either TLR1 or TLR6). In addition, TLR4 is capable of recognizing the *Mtb* secreted heat shock protein 60/65, while TLR9 recognizes unmethylated CpG motifs in mycobacterial DNA. Studies with genetically altered mice showed an increased susceptibility to *Mtb* infection in the absence of TLR2, TLR9 and possibly TLR4 [4,5]. In accordance, MyD88, an adaptor molecule utilized by most Toll/Interleukin-1 receptor (TIR) domain containing receptors, proved of particular importance for host defense against *Mtb*, although this seems mainly related to its role in interleukin (IL)-1 signaling [5,6]. Mycobacterial engagement of phagocyte TLRs and subsequent recruitment of MyD88 eventually culminates in translocation of NF-κB and a robust proinflammatory response. Aberrant TLR signaling may cause collateral tissue damage and dampening of the inflammatory response can help to limit detrimental immunopathological changes. Several negative regulators of innate immunity and TLR signaling have been identified, including cell-surface receptors, such as IL-1 receptor-like 1 (ST2) and single immunoglobulin IL-1R-related molecule (SIGIRR, TIR8), and intracellular inhibitors, such as Toll-interacting protein (TOLLIP), suppressor-of-cytokine signaling (SOCS) and IL-1 receptor associated kinase (IRAK)-M [7].

Several earlier studies reported on the expression of TLRs by blood leukocytes in patients with TB [8-11]. We here sought to determine expression of inhibitory regulators of innate immunity in the circulation and at the primary site of infection in patients with lung TB. In addition, we evaluated the value of soluble (s)ST2, a secreted protein produced by the *st2* gene [12], as a potential biomarker in TB.

## Methods

### Study design and population

Patients and healthy blood donors were recruited prospectively in the Tuberculosis Clinic of Chittagong General Hospital and the Chittagong Medical College & Hospital, Chittagong, Bangladesh. TB suspicion was based on the WHO-based National Guidelines for Bangladesh [13]. Two patient groups were studied. For the first group, from which only blood was drawn, inclusion criteria were: (a) 18–80 years of age; (b) confirmed pulmonary TB; (c) ability to give written informed consent prior to study-specific procedures. Exclusion criteria were: (a) a concomitant disease or a known clinical condition which could interfere with the conduct of the study; or (b) an unwillingness or inability to comply with the study protocol for any other reason. On-site TB confirmation was defined by a minimum of two out of three positive Ziehl-Neelsen (ZN)-stained sputum samples collected on two consecutive days. Subsequent confirmation of *Mtb* infection was obtained by PCR (GeneXpert, Cepheid, Solna, Sweden) in the Laboratory of Medical Microbiology in the Academic Medical Center (Amsterdam, the Netherlands) for all but two patients in which PCRs could not be performed due to technical difficulties. Healthy blood donors served as controls.

For the second patient group, in which diagnostic bronchoscopies were performed, inclusion criteria comprised: (a) 18–65 years of age; (b) clinical suspicion of pulmonary TB, according to the WHO-based National Guidelines for Bangladesh [13], (c) three consecutive ZN stained sputum samples negative for *Mtb*; (d) unilateral abnormalities on chest X-ray suspect for TB; (e) no TB treatment; (f) ability to give written informed consent prior to any study-specific procedure. Blood samples were taken directly at presentation followed by a bilateral bronchoalveolar lavage to obtain BAL fluid and BAL cells. *Mtb* infection was confirmed on site by a ZN-positive BAL stain or in Amsterdam by PCR. Prior to bronchoscopy the exact location of the diseased lung subsegment was identified by chest X-ray. Bilateral BALs were performed by qualified pulmonologists in a standardized fashion according to the guidelines of the American Thoracic Society, using a flexible direct bronchoscope (Olympus type C30C, P20D and P40, Shinjuku, Tokyo, Japan). Eight successive 20-mL aliquots of sterile saline 0.9% were instilled at the uninfected side in a subsegment of the middle lobe or lingula and aspirated immediately with low suction. This was followed by instillation and aspiration of the same amount of aliquots in the contralateral, diseased lung subsegment. Bronchoscopies were not performed in patients with ZN-positive sputum, since these would not contribute to the diagnosis and patient management.

All patients were tested for human immunodeficiency virus (HIV) infection by a Determine® HIV 1/2 test (Alere, Tilburg, The Netherlands). The study was approved by the National Research Ethics Committee (NREC), Bangladesh Medical Research Council, Bangladesh and the Oxford Tropical Research Ethics Committee, University of Oxford, Oxford, UK (OXTREC 35–09). Written informed consent was obtained from all study subjects or next-of-kin by a native Bengali speaker.

### Sample handling

Blood samples for plasma collection (EDTA and heparinized blood) and peripheral blood mononuclear cell (PBMC) harvesting (Cell Preparation Tubes (CPT), Becton Dickinson, Franklin Lakes, NJ) were taken directly at presentation. PBMCs were isolated according to the manufacturer’s instructions and preserved in RA1 lysis buffer for later RNA extraction (Nucleospin RNA kit, Macherey-Nagel, Dueren, Germany) or viably frozen (see flow cytometry) awaiting additional analysis. Alveolar macrophages (AMs) were isolated from BAL fluid using CD71 MACS beads (Miltenyi Biotec, Bergisch Gladbach, Germany) as described [14]. In brief, BAL fluid was centrifuged and cells were resuspended in ice-cold sterile automated magnetic cell sorting and separation (autoMACS) buffer (PBS, 0.5% bovine serum albumin, 2 mM EDTA; pH = 7.4). Subsequently, cells were incubated for 15 min with CD71 microbeads at 4°C. Cells were washed again in autoMACS buffer and purified by autoMACS (Multenyi Biotec). The average AM purity was 96% as determined on cytospins. AMs were preserved in RA1 lysis buffer for RNA analysis (Nucleospin RNA kit, Macherey-Nagel, Dueren, Germany).

### Assays

Levels of the following cytokines, chemokines and other inflammatory markers were measured in EDTA-plasma by multiplex assay (Luminex, Austin, TX) using reagents from Bio-Rad (Veenendaal, the Netherlands): interleukin (IL)-6, chemokine (C-X-C motif) ligand (CXCL) 8 (IL-8), soluble IL-2 receptor subunit-α (sIL-2Rα), soluble intercellular adhesion molecule 1 (sICAM-1), soluble TNF receptor-1 and −2 (sTNFR-1, sTNFR-2). IL-33 and sST2 were measured by specific enzyme-linked immunosorbent assays (R&D systems, Abingdon, UK). C-reactive protein (CRP) was measured in heparinized plasma samples with the C-Reactive Protein Gen.3 test kit (Roche Diagnostics, Mannheim, Germany), an immunoturbidimetric method, on the Hitachi Modular P-800 module (Hitachi, Hitachinaka, Japan).

### Flow cytometry

Freshly obtained PBMCs were frozen in RPMI medium with 20% FCS and 20% DMSO with the help of a ‘mr. Frosty’ freezing container (Thermo Scientific, Waltham, US) first placed overnight in a −20° freezer and subsequently stored in the gas phase of a liquid nitrogen container. Prior to analysis stored cells were carefully thawed, washed and stained with the following antibodies: CD14-APC-Cy7, CD3-Alexa Fluor 700, CD4-PERCP-Cy5.5 or CD4-PE (all BD Biosciences), CD8-PE-Cy7 (eBioscience), ST2-FITC (Acris, Herford, Germany) and SIGIRR-APC (R&D Systems) for 30 minutes at 4°C. To stain for TOLLIP cells were permeabilized with perm I buffer (BD Biosciences) for 20 minutes at 4°C. Permeabilized cells were stained intracellularly with antibodies against TOLLIP (SouthernBiotech, Birmingham, AL) for 30 minutes at 4°C followed by staining with a secondary anti-mouse-Alexa Fluor 610-R-Phycoerythrin (Life technologies, Carlsbad, CA) antibody for 30 minutes at 4°C. Data acquisition was performed using a FACS Canto II (BD biosciences) flow cytometer. Measured geomean fluorescence intensities (MFIs) were corrected with the help of the fitting fluorescence minus one (FMO) measurements. For cytometric analysis 17 (healthy donor) versus 19 (TB patient) were compared.

### Evaluation of mRNA levels by quantitative RT-PCR

Total RNA obtained from PBMCs and AMs was reverse transcribed using oligo(dT) primer and moloney murine leukemia virus reverse transcriptase (Promega, Madison, WI, USA) according to recommendations of the suppliers. RT-PCRs were performed using FastStart DNA Master SYBR Green I in a Light Cycler apparatus (Roche Applied Science, Penzberg, Germany). Primers used are indicated in Table 1. Quantitative PCR data were analyzed with the LinRegPCR program [15,16]. All samples were normalized to the house keeping gene β2-microglobulin [17,18].

**Table 1:** **Primer sequence used for real-time PCR using the Light Cycler**

**Gene**		**Primer sequence**
*IRAK-M*	Forward,	GTACATCAGACAGGGGAAACTTT
	Reverse,	GACATGAATCCAGGCCTCTC
*SIGIRR/ Tir8*	Forward,	CAGACCCATCTTCATCACCTTC
	Reverse,	GCTGCACTTCTTTCCAAAAATC
*A20*	Forward,	TCCAGAACACCATTCCGTG
	Reverse,	TGAGGTGCTTTGTGTGGTTC
*Tollip*	Forward,	CTGATGCCAACAGTGTACCAG
	Reverse,	ACATGTCCTGGATGGCTTTC
*ST2/ IL1RL1*	Forward,	TCAATAGGACTGGATATGCGA
	Reverse,	GCCCTGTACCTTGATCCTTG
*MKP-1*	Forward,	CAACCACAAGGCAGACATCA
	Reverse,	CTTCGCCTCTGCTTCACAA
*SOCS-1*	Forward,	AGAGCTTCGACTGCCTCTTC
	Reverse,	AGGGGAAGGAGCTCAGGTAG
*SOCS-3*	Forward,	CAGTCTGGGACCAAGAACCT
	Reverse,	GAGGAGGGYYCAGTAGGTGG
*β*_*2*_ *-microglobulin*	Forward,	CTCGCGCTACTCTCTCTTTCT
	Reverse,	TGCTCCACTTTTTCAATTCTCT

*Irak-m*: Interleukin (IL)-1 receptor (R) associated kinase-m, *Sigirr*: single immunoglobulin IL-1 related receptor (molecule), *Tir8*: Toll/IL-1R 8, *Tollip*: Toll interacting protein, *IL1RL1*: IL-1R like-1, *MKP-1*: Map kinase phosphatase-1, *Socs*: suppressor of cytokine signaling.

### Statistical analysis

Data are expressed as dot plots with medians (figures) or medians with interquartile ranges (tables). Comparisons between groups were performed using the Mann–Whitney *U* test. Comparisons between paired samples were performed using a Wilcoxon matched pairs test. Analyses were done using Graphpad Prism version 5.01 (San Diego, CA). Correlations were calculated using the Spearman rho test via SPSS version 16.0 (Armonk, NY). *P*-values < 0.05 were considered statistically significant.

## Results

### Patient characteristics

Two patient groups with lung TB were studied. The first group, from which plasma and PBMCs were obtained, consisted of 54 patients with primary TB, who were compared with 29 healthy blood donors. The second group, consisting of patients suspected to have lung TB but with ZN negative sputum, comprised 23 patients, 9 of whom were confirmed to have TB by *Mtb* specific PCR on BAL material. Patient characteristics and clinical features are summarized in Table 2.

**Table 2:** **Patient characteristics**

	**Systemic host response**		**Local host response**
	**Healthy controls** ***n*** **= 29**	**Primary TB** ***n*** **= 54**	**All TB-positive bronchoscopy patients** ***n*** **= 9**
*Demographics*			
Age (years)	31 (26–35)	28 (22–42)	35 (24–48)
Male sex (*n, %*)	21 (72%)	40 (74%)	7 (78%)
Smoker (*n, %*)	8 (28%)	27 (50%)	5 (56%)
HIV-positive (*n, %*)	0 (0%)	1 (2%)	1 (11%)
*Symptoms*			
Fever (*n, %*)	0 (0%)	54 (100%)	9 (100%)
Night sweats (*n, %*)	0 (0%)	21 (39%)	1 (11%)
Weight loss (*n, %*)	0 (0%)	39 (72%)	4 (44%)
Fatigue (*n, %*)	1 (3%)	29 (54%)	4 (44%)
Shortness of breath (*n, %*)	0 (0%)	6 (11%)	0 (0%)
Productive cough (*n, %*)	2 (7%)	50 (93%)	8 (89%)
*Signs*			
Temperature (°C)	36.9 (36.5-37.1)	37.4 (36.8-38.1)***	37.3 (36.5-38.3)*
MAP (mmHg)	83.3 (80–93.3)	80 (70–87)**	77 (73–82)**
Heart rate (bpm)	80 (78–84)	90 (81–100)***	84 (80–105)
Respiratory rate (brpm)	20 (20–24)	25 (24–28)***	20 (20–28)
BMI (w/l^2^)	24 (22.4-25.7)	17.7 (15.6-19.6)***	18.3 (16.3-22.6)**

Abbreviations: *TB* tuberculosis, *n* number of patients, *MAP* mean arterial blood pressure, *bpm* beats per minute, *brpm* breaths per minute, *BMI* body mass index, expressed as weight (w) divided by length (l)^2^. Percentages given are within study groups. Data presented are medians with interquartile ranges. Mann Whitney-U test: *P < 0.05, **P < 0.01, ***P < 0.001 when compared to healthy controls.

### Expression of cell-surface negative regulators of innate immunity and plasma sST2 concentrations in patients with active TB

**Figure 1:**
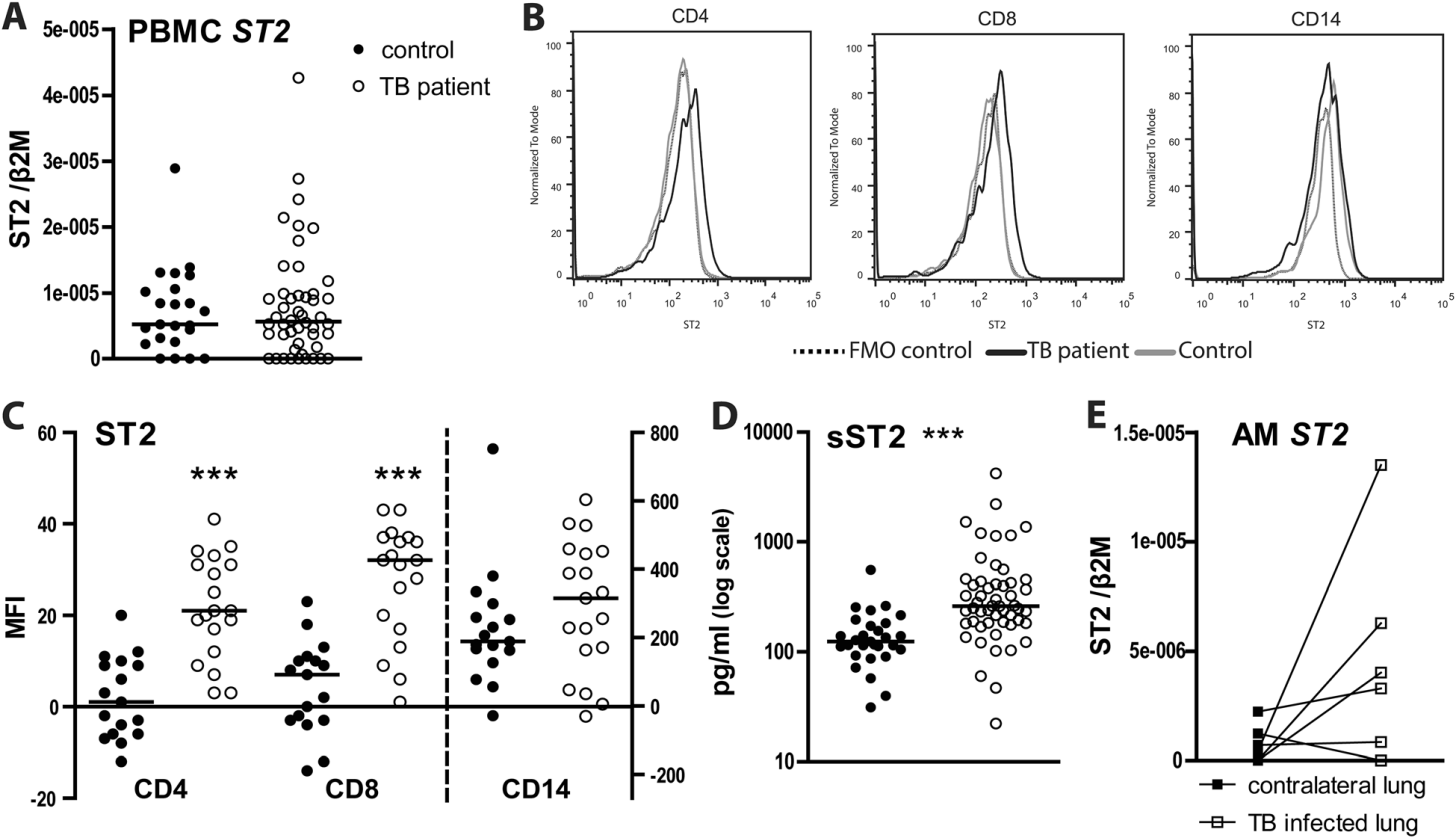
**ST2 during active pulmonary tuberculosis. (A)** ST2 mRNA in PBMCs; **(B)** ST2 surface expression measured on CD4 and CD8 positive lymphocytes and CD14 positive monocytes (representative histograms). FMO, fluorescence minus one. **(C)** Idem for individual subjects (geomean channel fluorescence intensity (MFI)). **(D)** Plasma sST2 concentrations. **(E)** ST2 mRNA in alveolar macrophages (AM) of TB patients originating from the TB infected lung (open squares) or contralateral lung (closed squares). Depicted mRNA levels are normalized to the house keeping gene β2-microglobulin. Depicted are dot plots with medians; open dots: TB patients, closed dots: healthy donors. ***P < 0.001.

ST2 and SIGIRR are cell-surface associated receptors that have been shown to inhibit TLR signaling [19,20]. ST2 mRNA levels were low in PBMCs of both TB patients and healthy controls and not different between groups (Figure 1A). Flow cytometry revealed an upregulation of ST2 expression on CD4 and CD8 positive lymphocytes, but not on CD14 positive monocytes, of TB patients when compared to healthy controls (Figure 1B,C). Patients with lung TB showed elevated plasma levels of sST2 (261 [183–430] pg/ml; median [IQR]) relative to healthy controls (124 [99–177] pg/ml; *P* < 0.001) (Figure 1D). In contrast, the plasma levels of the ST2 ligand IL-33 were below or just above the detection limit in both patients and controls and not different between groups (data not shown). We next investigated whether plasma sST2 levels correlated with the plasma concentrations of established biomarkers of TB. For this we measured IL-6, IL-8 and IP-10 (which are elevated in active TB), as well as sIL-2Rα, sICAM-1, sTNFR-1, sTNFR-2 and CRP (which correlate with the extent of disease in TB) [21] in plasma of TB patients and healthy controls (Table 3). As expected [21], the plasma levels of all biomarkers were elevated in TB patients (all *P* < 0.001 versus controls). Plasma sST2 correlated significantly with the plasma levels of these TB biomarkers, although correlations were relatively modest (Table 4). The strongest correlations were found with plasma sIL-2Rα (*r* = 0.52, *P* < 0.001) and IL-8 (*r* = 0.50, *P* < 0.001).

**Table 3:** **Parameters of systemic inflammation during pulmonary TB**

	**Controls** ***n*** **= 29**	**TB** ***n*** **= 54**
**IL-6 (pg/mL)**	0.3 (0.06-0.7)	5.8 (3.3-12)***
**IL-8 (pg/mL)**	0.8 (0.4-1.4)	2.2 (1.3-3.7)***
**IP-10 (ng/mL)**	0.37 (0.3-0.46)	2.8 (1.6-3.7)***
**sIL-2Rα (pg/mL)**	74 (54–101)	225 (152–349)***
**sICAM-1 (ng/mL)**	170 (145–214)	344 (279–418)***
**sTNFR-1 (ng/mL)**	0.9 (0.7-1.0)	1.7 (1.1-2.0)***
**sTNFR-2 (ng/mL)**	5.1 (4.6-6.0)	10 (7.8-13)***
**CRP (μg/mL)**	0.9 (0.3-2.6)	51 (23–90)***

Abbreviations: IL interleukin; IP-10 Interferon gamma-induced protein (CXCL) 10; sIL-2Rα soluble interleukin-2 receptor α; sICAM-1 soluble intercellular adhesion molecule-1; sTNFR-1,-2 soluble tumour necrosis factor receptor-1 and −2; sST2 soluble ST2 (IL1R like 1); CRP C-reactive protein. Data are medians with interquartile ranges. ***P < 0.001 for the difference between primary TB versus controls. Depicted are medians with interquartile ranges.

**Table 4:** **Correlations of sST2 with TB biomarkers in patients with primary lung TB**

		**CRP**	**IL-6**	**IL-8**	**IP-10**	**sIL-2Rα**	**sICAM-1**	**sTNFR-1**	**sTNFR-2**
**sST2**	***r***_s_	0.32	0.33	0.50	0.43	0.52	0.43	0.37	0.39
	***P***	0.02	0.02	<0.001	0.001	<0.001	0.001	0.006	0.004

Abbreviations: IL interleukin; IP-10 Interferon-γ induced protein (CXCL-10); sIL-2R soluble interleukin-2 receptor; sICAM soluble intercellular adhesion molecule; sTNFR soluble tumour necrosis factor receptor; CRP C-reactive protein; *r*_s_ Spearman rank coefficient of correlation adjusted for multiple comparisons using Bonferroni’s procedure.

To obtain insight in (s)ST2 expression at the site of the infection, we performed bilateral BAL in 9 patients with confirmed TB and harvested AMs from the site of infection (as determined by chest X ray) and from the contralateral lung (not showing a radiologic infiltrate). sST2 was not detectable in BAL fluid of patients with lung TB. In addition, ST2 mRNA levels did not differ in AMs from the infected side and the contralateral lung (Figure 1E).

**Figure 2:**
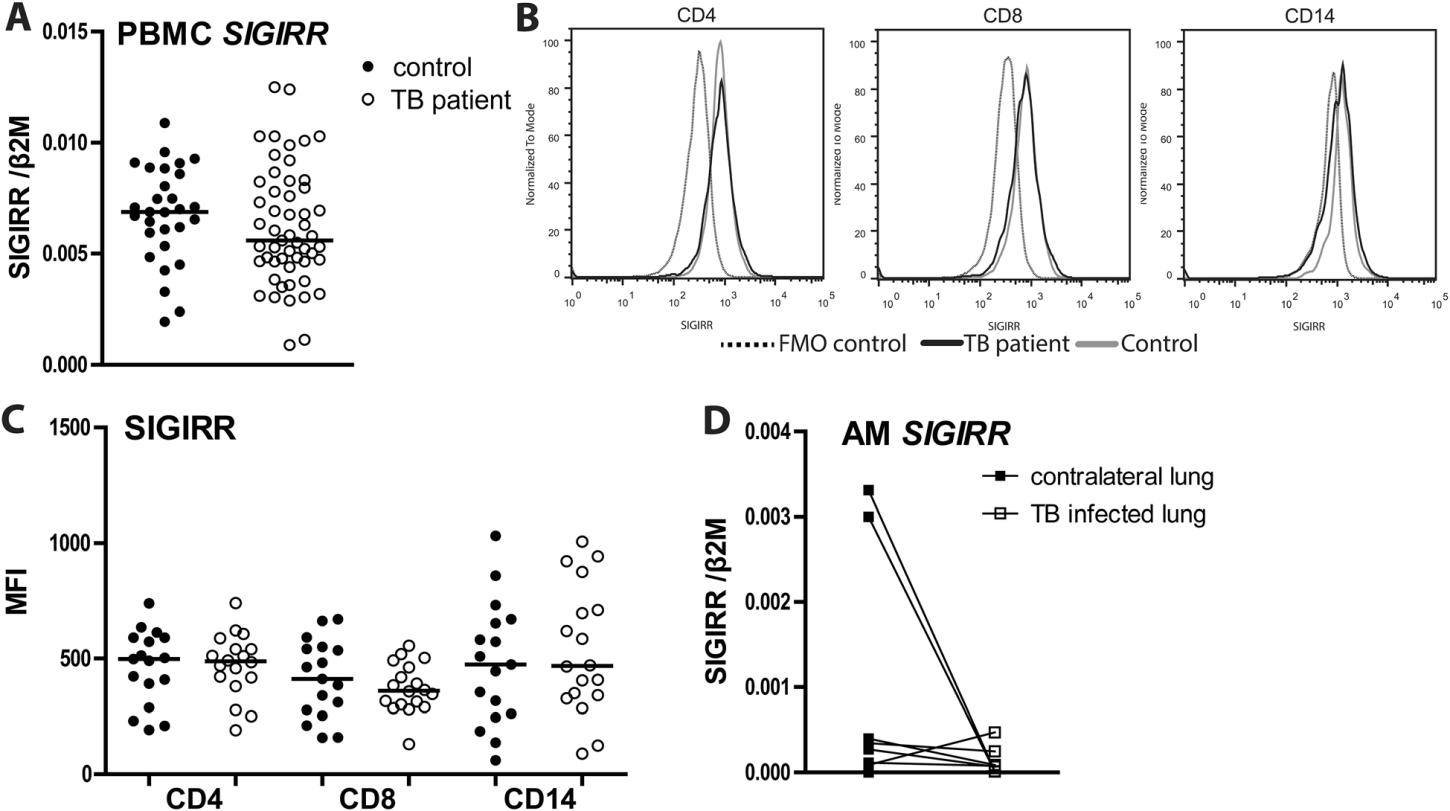
**SIGIRR during active pulmonary tuberculosis. (A)** SIGIRR mRNA in PBMCs. **(B)** SIGIRR surface expression measured on CD4 and CD8 positive lymphocytes and CD14 positive monocytes (representative histograms). FMO, fluorescence minus one. **(C)** Idem for individual subjects (mean channel fluorescence intensity, MFI). **(D)** SIGIRR mRNA in alveolar macrophages (AM) of TB patients originating from the TB infected lung (open squares) or contralateral lung (closed squares). Depicted mRNAs are normalized to the house keeping gene β2-microglobulin. Depicted are dot plots with medians; open dots: TB patients, closed dots: healthy donors.

SIGIRR mRNA levels did not differ in PBMCs from patients and controls (Figure 2A). In addition, flow cytometry showed similar expression levels in CD4 positive and CD8 positive lymphocytes, and on CD14 positive monocytes (Figure 2B,C). SIGIRR mRNA levels did not differ in AMs from the infected side and the contralateral lung (Figure 2D). Overall these data indicate that SIGIRR expression is not altered during TB.

### Expression of intracellular negative regulators of innate immunity in patients with active TB

**Figure 3:**
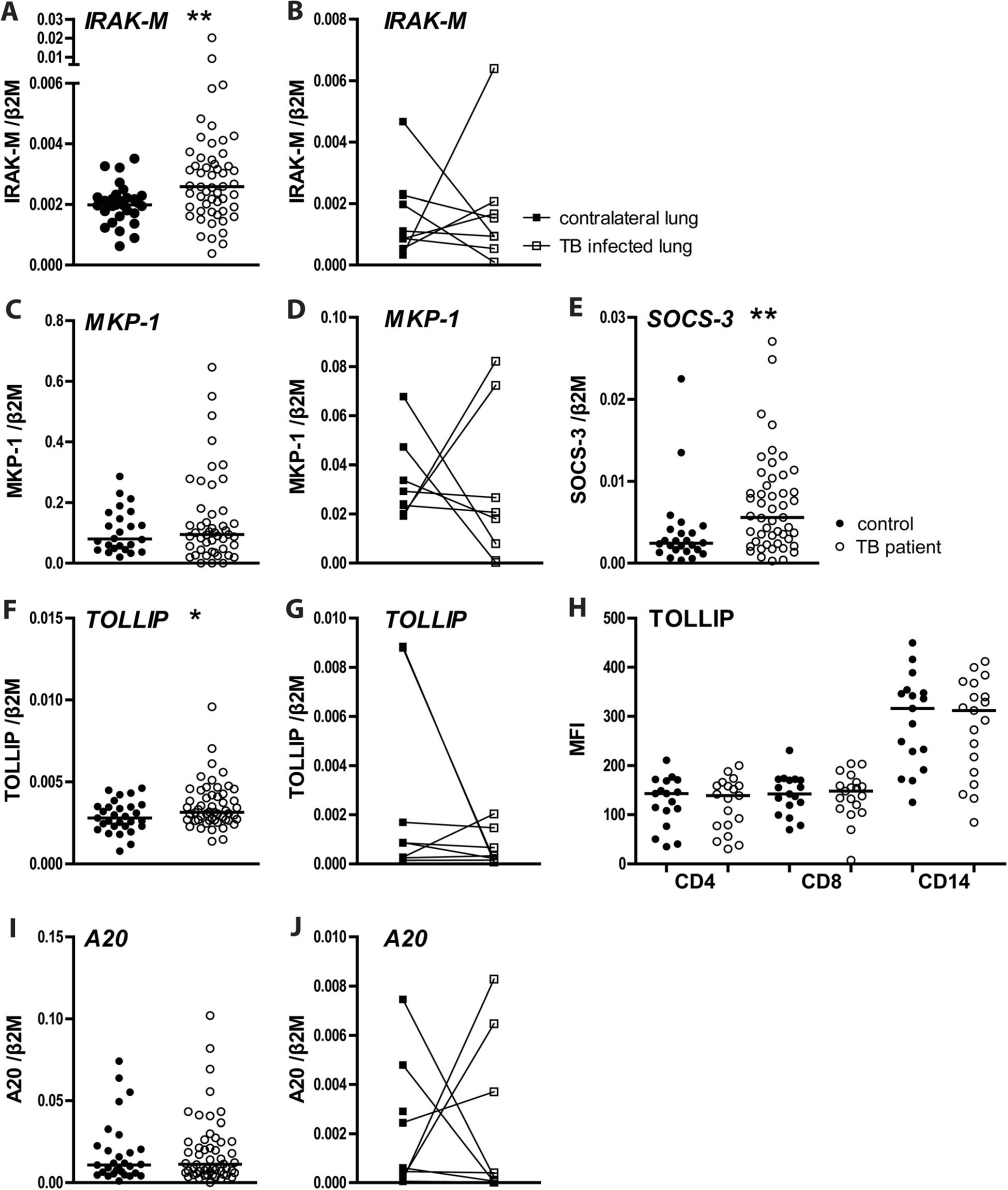
**Intracellular negative regulators and mediators of TLR signaling.** mRNA levels in PBMCs of TB patients (open dots) and healthy controls (closed dots) and TB patient AMs from the diseased (TB-positive) lung segment (open squares) and from the matching contralateral lung (closed squares). PBMC mRNA expression of IRAK-M **(A)**, MKP-1 **(C)**, SOCS-3 **(E)**, TOLLIP **(F)** and A20 **(I)**. AM mRNA expression of IRAK-M **(B)**, MKP-1 **(D)**, TOLLIP **(G)** and A20 **(J)**. TOLLIP **(H)** expression levels in CD4 or CD8 positive lymphocytes and CD14 positive monocytes, as determined by flow cytometry (mean channel fluorescence intensity, MFI). mRNA expression is normalized to β2-microglobulin. Depicted are dot plots with medians; open dots: TB patients, closed dots: healthy donors. *P < 0.05, **P < 0.01.

We next sought to determine the expression of intracellular negative regulators of innate immunity in the blood compartment of TB patients (Figure 3). TB patients showed higher levels of mRNAs encoding IRAK-M (*P* <0.01 versus controls), TOLLIP (*P* <0.05) and SOCS-3 (*P* <0.01), whereas the levels of mRNAs encoding A20 and mitogen-activated protein kinase phosphatase (MKP)-1) did not differ between groups. SOCS-1 mRNA could not be detected in PBMCs of either TB patients or controls. The extent of IRAK-M, TOLLIP or SOCS-3 mRNA expression did not correlate with the plasma concentrations of CRP, IL-6, IL-8, IP-10, sIL-2Rα, sICAM-1, sTNFR1 or sTNFR2 (data not shown). Flow cytometry showed no difference in TOLLIP expression between PBMCs from TB patients and healthy controls. mRNA levels of IRAK-M, MKP-1, A20 or TOLLIP did not differ in AMs from the infected site and the contralateral lung (Figure 3). SOCS-1 and −3 mRNAs could not be detected in AMs.

## Discussion

TLRs are important players in the recognition of mycobacterial pathogens and the facilitation of the antimicrobial immune response [4,5]. Negative regulators of TLR signaling dampen TLR induced inflammation [7], which on the one hand can prevent detrimental pathological changes but on the other hand can provide a pathway for *Mtb* immune evasion. Here we sought to obtain insight in the expression of negative innate immunity and TLR regulators in the blood compartment and at the site of infection in patients with TB from an endemic region in Bangladesh. We showed differential expression of several inhibitory regulators of innate immunity at either mRNA or protein level between patients with active TB and healthy controls and identified plasma sST2 as a potential novel biomarker for TB, correlating with established biomarkers of TB disease activity.

Many previous studies examined the role of TLRs in TB. Among the TLR family, TLR2, TLR4 and TLR9 have been implicated in host defense against *Mtb*, although the impact of deficiency of either one of these receptors was modest or variable in mouse TB models [5,6]. Certain gene polymorphisms in TLRs and related genes (*i.e.*, TLR1 [22], TLR2 [23-25], and MyD88-adaptor-like (MAL) [25,26]) have been associated with susceptibility to TB in humans, suggesting that TLR signaling influences TB development in the human host. Patients with active pulmonary TB showed increased TLR1, 2, 4 and 6 mRNA levels in whole blood leukocytes when compared with healthy controls, while TLR 7 and 9 mRNA expression was unaltered [9].

ST2 inhibits MyD88 dependent signaling [19], and, together with IL-1R accessory protein, functions as the IL-33 receptor [27]. In addition, a sST2 variant exists which can act as a decoy receptor for IL-33 [12]. We here detected elevated levels of sST2 in plasma of TB patients. Elevated circulating sST2 levels have been reported in different human disease settings, including auto-immune diseases like systemic lupus erythematosus and rheumatoid arthritis [28], asthma exacerbations [29] and cardiovascular disease [30]. Our laboratory previously reported elevated sST2 levels in sepsis [31] and leptospirosis [32]. Hence, elevated sST2 plasma levels by no means are specific for TB. Rather, circulating sST2 levels could be a marker for disease activity in TB patients, also considering the statistically significant correlations between sST2 and known TB biomarkers [21]. Clearly, the potential value of sST2 as a TB biomarker and its response to TB treatment need to be tested further in independent cohorts. We only detected very low amounts of ST2 mRNA in PBMCs of patients with TB. In accordance, our laboratory previously reported a marked rise in plasma sST2 concentrations after intravenous administration of LPS to healthy humans in the absence of an increase in ST2 mRNA levels in blood leukocytes [17]. We also found equally low ST2 mRNA levels in AMs from lungs of TB patients. In agreement, Oshikawa *et al.* tested different lung macrophages for ST2 expression (human and murine cell-lines, and primary human AMs) and found low or undetectable mRNA levels unless cells had been exposed to LPS, IL-1β, TNF-α or IL-6 [29]. Importantly, our group previously documented that stimulation of whole blood with LPS or viable *Leptospira* does not result in sST2 release [32]. Together these results indicate that plasma sST2 originates from cells not present in blood. Interestingly, we found increased ST2 surface expression on blood lymphocytes of TB patients, although ST2 expression was low relative to that on monocytes. Of note, ST2 deficiency did not influence mycobacterial loads or disease outcome during murine *Mtb* infection [33]. Plasma IL-33 was not elevated in patients with TB. In accordance, children with TB showed unaltered circulating IL-33 levels [34].

SOCSs are a family of intracellular proteins functioning as feedback inhibitors of cytokine receptors. They are induced by TLR activation and either directly (SOCS-1) or indirectly (SOCS-3) interfere with TLR signaling [35]. While we could not detect SOCS-1 mRNA in PBMCs, confirming a previous report from our group [17], we report increased mRNA expression of SOCS-3 in PBMCs from pulmonary TB patients when compared to PBMCs from healthy controls. In contrast, Masood *et al.* reported increased SOCS-1 mRNA expression in PBMCs from patients with advanced lung TB in the absence of changes in SOCS-3 mRNA expression [36,37]. In accordance with our current data, SOCS-3 mRNA expression was reported increased in whole blood leukocytes [38]. Another investigation demonstrated enhanced mRNA SOCS-1 and SOCS-3 mRNA levels in induced sputum from TB patients [8]. We were not able to detect either SOCS-1 or −3 mRNA in AMs from TB patients suggesting that SOCS-1 and −3 expression in induced sputum might originate from a different cell type. A recent study has established the important functional role for SOCS-3 expression in either lymphoid or myeloid cells for resistance against *Mtb* in mice [39].

To the best of our knowledge our study is the first to report increased IRAK-M mRNA expression in PBMCs from TB patients. It has been suggested that *Mtb* lipoarabinomannan can induce IRAK-M in macrophages, thereby attenuating pro-inflammatory signals [40]. Indeed, IRAK-M mRNA expression was upregulated in induced sputum from TB patients [8]. We did not find increased IRAK-M mRNA levels in AMs from TB patients. Of note, however, control AMs were obtained from the contralateral lung of the same patient. Although patients were selected for one sided radiographic abnormalities and although the contralateral BAL fluids tested PCR negative for *Mtb*, one cannot rule out a spillover effect from the affected to the contralateral lung. Furthermore, ethical considerations prevented us from recruiting sputum smear positive patients for BAL procedure, meaning that bacterial loads in these patients were low enough to escape repetitive sputum smear testing (in contrast to the pulmonary TB patients recruited for PBMC analysis). Therefore, it is possible that in patients with more extensive pulmonary TB, gene expression is altered in AMs harvested from the site of infection.

In addition to elevated SOCS-3 and IRAK-M mRNA levels we observed slightly enhanced expression of mRNA encoding TOLLIP in PBMCs of TB patients, which, however, was not accompanied by enhanced protein expression. In resting cells TOLLIP forms a complex with either IRAK-1 or −2 thereby preventing IRAK (auto)phosphorylation and inhibiting IL-1R, TLR2 and TLR4 signaling pathways [41,42]. In accordance, human peripheral blood monocytes in which TOLLIP was partially silenced produced more TNFα and IL-6 upon stimulation with *Mtb* whole cell lysates [43]. Although this might suggest that TOLLIP may hamper the antimycobacterial immune response, SNPs in the human *tollip* gene resulting in deficiency are associated with an increased susceptibility to TB [43]. As such, the functional role of TOLLIP in TB has yet to be determined. Notably, the extent of TOLLIP (and IRAK-M) mRNA expression in PBMCs did not correlate with circulating levels of proinflammatory cytokines. This does not contradict the assumption that the enhanced expression of negative innate immunity regulators serves to inhibit aberrant inflammation caused by activation of immune enhancing receptors such as TLRs. Rather, it is the balance between pro- and anti-inflammatory regulators that determines the net effect on proinflammatory cytokine release.

MKP-1 inactivates MAPKs (in particular p38 and JNK) which are downstream of TLR signal transduction pathways [44]. Silencing of MKP-1 in human blood monocytes decreased phospho-MAPK expression and TNF-α production in response to BCG, suggestive for a possible detrimental role of MKP-1 in host defense against *Mtb* [45]. We here did not find an effect of active TB on MKP-1 mRNA expression in either PBMCs or AMs. Similarly, TB did not influence mRNA levels of A20 or SIGIRR in PBMCs or AMs. A possible role for A20 in TB pathogenesis has not been investigated thus far, although its central role as an inducible inhibitor of NF-κB activation suggests A20 may be important [46]. Consistent with the TLR inhibiting role of SIGIRR, SIGIRR deficient mice showed exaggerated inflammation and increased lethality after infection with *Mtb* despite an efficient control of mycobacterial growth [47].

## Conclusions

Innate immunity needs to be controlled carefully in order to prevent damage to cells and tissues due to aberrant inflammation. The immune system harbors a large variety of negative regulators that upon interaction between immune cells and pathogens become activated in parallel with immune enhancers. While previous studies examined the expression of immune enhancers, and in particular TLRs, during TB [8-11], we here report on an expression of a group of inhibitory regulators of innate immunity, differential expression of these “immune controllers” in PBMCs of patients with active TB relative to healthy blood donors. As such, our study provides evidence that in patients with TB activation of innate immunity is kept in check by several negative regulators. Furthermore, in patients elevated sST2 plasma levels correlated with several established TB biomarkers, suggesting that sST2 could be a useful marker for TB disease activity and treatment response.

## Abbreviations

AM: Alveolar macrophage
BAL: Bronchoalveolar lavage
CRP: C-reactive protein
CXCL-8: Chemokine (C-X-C motif) ligand 8 (IL-8)
FMO: Fluorescence minus one
IL: Interleukin
IP-10: Interferon-γ-induced protein (CXCL) 10
IRAK: IL-1 receptor associated kinase
IQR: Interquartile range
MAL: MyD88-adaptor-like
MFI: Geomean fluorescence intensity
MKP: Mitogen-activated protein kinase phosphatase
*Mtb*: *Mycobacterium tuberculosis*
PBMC: Peripheral blood mononuclear cells
SIGIRR: Single immunoglobulin IL-1 receptor related molecule (TIR8)
SOCS: Suppressor-of-cytokine signaling
ST2: IL-1 receptor-like 1
sST2: Soluble ST2
sICAM-1: Soluble intercellular adhesion molecule 1
sIL-2Rα: Soluble IL-2 receptor subunit-α
sTNFR: Soluble tumor necrosis factor receptor
TB: Tuberculosis
TIR: Toll/IL-1 receptor
TLR: Toll-like receptor
TOLLIP: Toll- interacting protein
ZN: Ziehl-Neelsen
